# Thoracic Segmental Spinal Anesthesia for Upper Thoracic Spine Fractures: A Case Series

**DOI:** 10.7759/cureus.87861

**Published:** 2025-07-13

**Authors:** Richa Chandra, Luiz E Imbelloni, Imran Ahmed Khan, Anmol Singh

**Affiliations:** 1 Anesthesiology, Rohilkhand Medical College and Hospital, Bareilly, IND; 2 Anesthesiology, Independent Researcher, Rio de Janeiro, BRA; 3 Community Medicine, KMC Medical College and Hospital, Maharajganj, IND; 4 Anesthesiology, Adesh Institute of Medical Sciences and Research, Bathinda, IND

**Keywords:** comorbidities, high-risk patients, regional anesthesia, spinal cord, thoracic segmental spinal anesthesia, upper thoracic spine fractures

## Abstract

Unstable upper thoracic spine (T1-T6) fractures pose a considerable challenge in their management. These cases are often complicated by associated injuries. These types of thoracic fractures need early stabilization and surgical fixation, which have been shown to improve recovery and accelerate neurological outcomes.

Traditional general anesthesia (GA) with endotracheal intubation carries risks of postoperative pulmonary complications in patients with chest injuries or comorbidities. Spinal anesthesia (SA) has advantages over GA in terms of decreased blood loss, early enhanced recovery, and reduced risk of hazards associated with the prone position. Thoracic segmental SA (TSSA), which involves the targeted administration of local anesthetic near the surgical site, provides effective pain relief and allows for awake prone positioning without intraoperative complications or the need for conversion to GA.

This case series explores the feasibility and safety of TSSA for the surgical fixation of unstable upper thoracic spine fractures in five patients with significant comorbidities, including chest injuries and cardiovascular or respiratory issues. Postoperative courses were uneventful, with no neurological deterioration or need for mechanical ventilation at follow-up (13-30 months). These preliminary findings suggest TSSA as a potential alternative to GA for high-risk patients undergoing upper thoracic spine surgery, but larger, controlled trials are needed to establish its eﬀicacy and safety.

## Introduction

Unstable upper thoracic spine (T1-T6) fractures are challenging clinical conditions. These injuries are often caused by high-energy trauma such as automobile accidents, falls from heights, or sudden impacts from a moving projectile. They are mostly associated with chest injuries (such as hemothorax, pneumothorax, and rib fractures) as well as varying degrees of head and limb injuries [1]. Patients may present with complete or partial and often irreparable transection of the spinal cord, resulting in complete paraplegia, where surgery may need to be performed mainly for facilitation of palliative nursing care [2]. Early stabilization and fixation of unstable thoracic fractures improve recovery and accelerate neurological outcomes, preventing further damage [3,4].

Fractures in the transitional zone, such as those in the lower thoracic and upper lumbar spine, are more common than those in the upper thoracic spine from T1 to T6. This is because the thoracic spine in this region is fixed and stabilized by the rigid rib cage, sternum, and robust upper chest musculature [1,2]. Traumatic chest pathologies, such as hemothorax and rib fractures, cause significant pain during respiration, leading to limitation of respiratory effort and antecedent atelectasis. This may be followed by the formation of a consolidation and pneumonia. Additionally, the injured underlying lung can cause hypoventilation, hypercarbia, and ventilation/perfusion (V/Q) mismatch [5].

These fractures are challenging to both treating surgeons and anesthesiologists, as associated chest injuries make patients high-risk candidates for anticipated elective mechanical ventilation during the postoperative period. For years, the choice of anesthesia has been limited to general anesthesia (GA) with endotracheal intubation, especially for these high-risk subsets of patients. However, a few studies suggest that spinal anesthesia (SA) may be superior to GA in terms of decreased blood loss, early enhanced recovery, and reduced risk of hazards associated with the prone position. Yet, all studies are limited to fixation of the lumbar spine [6-8].

Recently, the increasing adaptation of thoracic segmental SA (TSSA) has changed the scenario [9]. Unlike lumbar SA, TSSA enables anesthesia for upper thoracic and truncal surgeries with lower doses of local anesthetics (LAs), minimizing motor impairment [10]. This method allows for a small, patient- and lesion-specific dose of LAs to create a dense dermatomal block at and around the surgical site. This case series explores the feasibility and safety of TSSA for the surgical fixation of unstable upper thoracic spine fractures in five patients with significant comorbidities, including chest injuries and cardiovascular or respiratory issues.

## Case presentation

We present a case series of five patients with unstable upper thoracic spine fractures managed under TSSA for surgical fixation between November 2022 and March 2024. Patient selection criteria included high-risk status (American Society of Anesthesiologists (ASA)-Physical Status III-IV), chest injuries, or cardiopulmonary comorbidities precluding safe GA and minimal spinal cord involvement. Exclusion criteria included coagulopathy, spinal cord compression, or refusal of regional anesthesia. Written informed consent was obtained from all subjects and/or a legal surrogate. Table 1 summarizes patient characteristics.

**Table 1:** Demographic and clinical characteristics of the participants T2DM: type 2 diabetes mellitus; CAD: coronary artery disease; CHF: chronic heart failure; CKD: chronic kidney disease; NYHA: New York Heart Association; RTA: road traffic accident; RWMA: regional wall motion abnormality; MR: mitral regurgitation; T: thoracic; ICD: intercostal drainage; PTCA: percutaneous transcatheter coronary angioplasty; HTN: hypertension; LL: lower limb; L: left; R: right; COPD: chronic obstructive pulmonary disease

Participants’ characteristics	Case 1	Case 2	Case 3	Case 4	Case 5
Age	65	45	80	32	44
Gender	Male	Male	Female	Male	Male
Comorbidities	Smoker, MR, RWMA, COPD	Uncomplicated balloon mitral valvulotomy, on prophylactic anticoagulation, HTN	T2DM, CAD, CHF, PTCA, CKD	Significant dyspnea on exertion, classified as NYHA class 2, obesity	HTN
Mechanism of injury	Fall from height	RTA	Falling at home while bathing	Fall from height	RTA
Diagnosis	Traumatic collapse of the T4 and T6 vertebrae	T8–T9 fracture	T3–T6 fracture	T6–7 fracture	T4 fracture
Neurological status at presentation	Power LL, R–1/5, L–2/5; sensation to pinprick from T4	LL–no motor power; patchy sensory level from T4/5	Complete paraplegia	LL R–2/5, L–3/5; sensation to touch present throughout	LL–R 0/5, L–1/5; paresthesia throughout
Planned surgery	Pedicle screw fixation of T3 to T7	T7–T10 fixation	T2–6 fixation	T5–8 fixation	T3–5 fixation
Associated injury	Multiple rib fractures, with hemothorax on the left side	Bilateral insertion of an ICD due to hemopneumothorax	Fracture of both bones in the left forearm	Multiple rib fractures	Left ICD

Case 1

**Figure 1:**
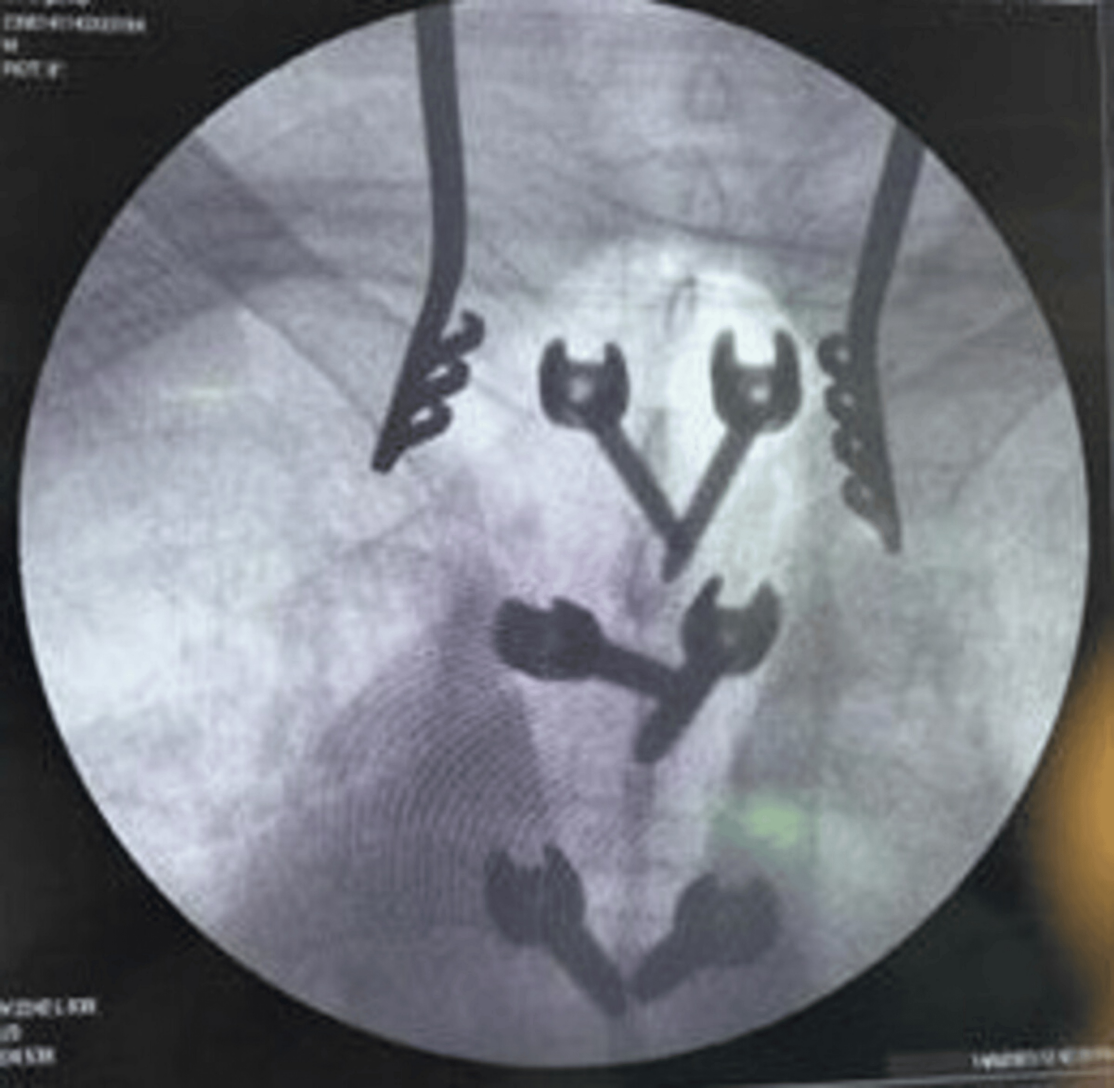
Intraoperative image (Case 1)

A 65-year-old man presented with a history of falling from a height, resulting in traumatic collapse of the T4 and T6 vertebrae. He had multiple rib fractures with a hemothorax on the left side, for which an intercostal drain (ICD) was placed. The patient was a smoker with a history of chronic obstructive pulmonary disease (COPD) and received irregular treatment. He was hemodynamically stable, his respiratory rate was 22 per minute, and peripheral oxygen saturation (SpO₂) was 88%-89% on room air. Auscultation revealed decreased air entry bilaterally, more pronounced on the left side. Administration of 4 L per minute of oxygen via a face mask improved his SpO₂ to 92%-93%. On incentive spirometry, he was able to lift a single ball. The electrocardiogram (ECG) showed old ischemic changes in the anterolateral leads, which corroborated with echocardiographic (ECHO) findings of a regional wall motion abnormality in the anterolateral wall at the mid-cavitary level, with a preserved ejection fraction of 50%. This was accompanied by a mild mitral regurgitation. The case was scheduled for pedicle screw fixation of the thoracic vertebrae from T3 to T7 on the fourth day of admission after optimizing his chest condition (Figure 1). A multidisciplinary approach was used, including a chest physician, an operating surgeon, and an anesthesiologist along with a chest physiotherapist.

Case 2

**Figure 2:**
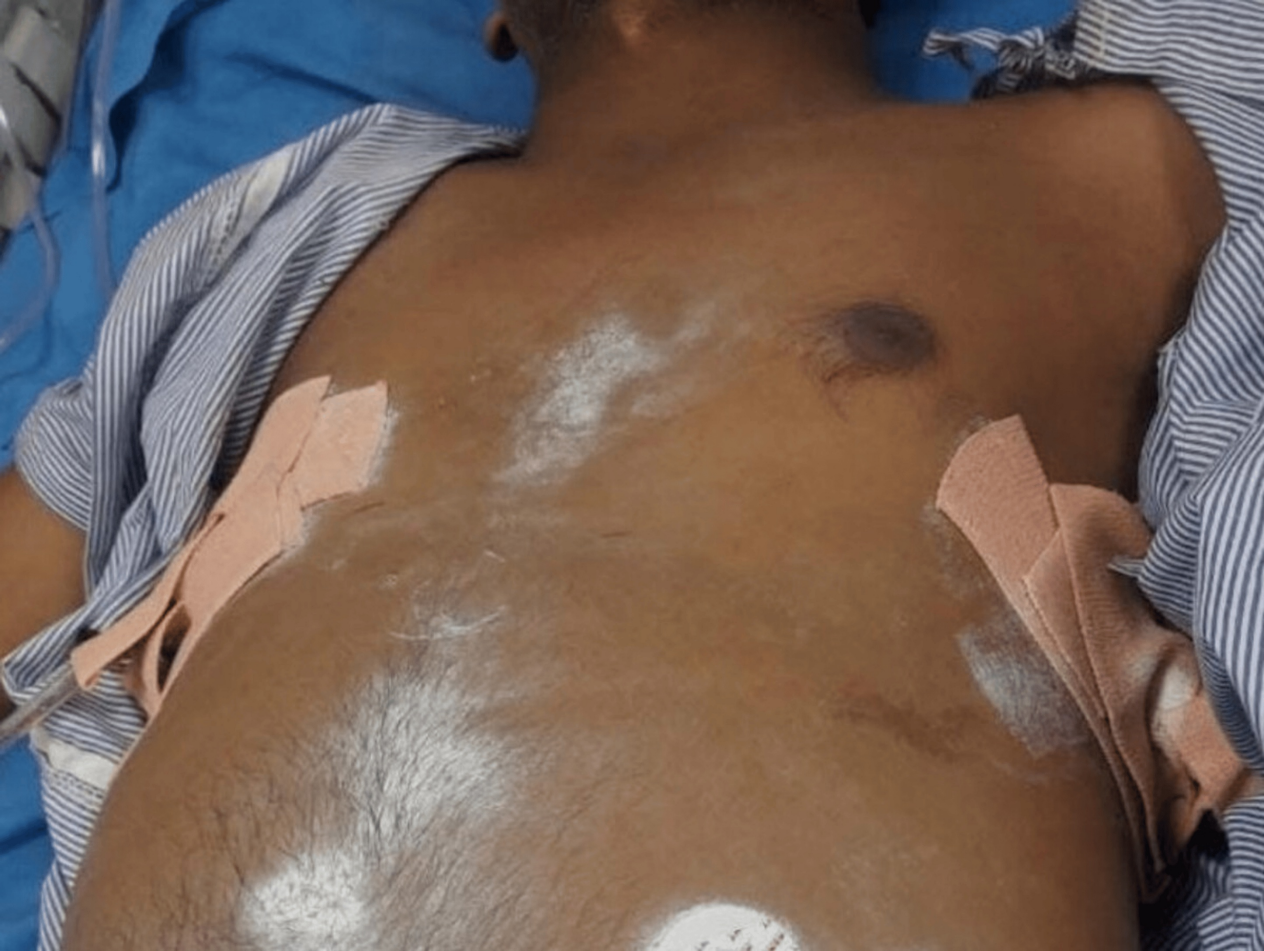
Bilateral intercostal drainage tube (Case 2)

A 45-year-old man presented with a history of a road traffic accident (RTA) with Modified Medical Research Council (MMRC) grade 2 dyspnea, accompanied by coughing and severe back pain, along with numbness in the lower scapular area, more pronounced on the right side. The patient had undergone bilateral insertion of an ICD due to hemopneumothorax (Figure 2). His SpO₂ was 94% on room air. Additionally, he also had a history of a successful balloon mitral valvulotomy for rheumatic heart disease seven years ago and was on prophylactic anticoagulation with a vitamin K inhibitor and penicillin. The patient had atrial fibrillation, with a controlled ventricular rate managed by metoprolol 50 mg orally once a day. The anticoagulation was stopped, and heparin bridging was initiated before the procedure.

Case 3

**Figure 3:**
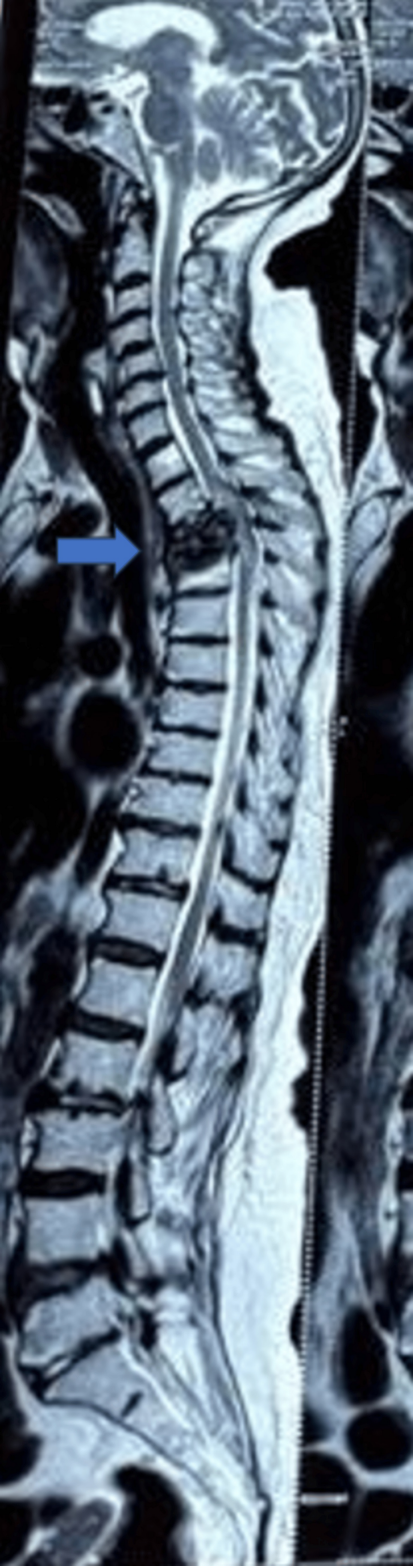
Case 3 showing fracture at T3–T6 The blue arrow shows the area of interest

An 80-year-old woman presented with a history of a fall at home while bathing, resulting in a fracture of both bones in the left forearm along with severe acute-onset mid-back pain and a burning sensation in the abdominal region. She received a cast for the forearm fracture. On investigating further, she was found to have a spine fracture and was scheduled for fixation of the T2-T6 vertebrae (Figure 3). She had type 2 diabetes mellitus, coronary artery disease, and chronic heart failure with moderately reduced ejection fraction, managed with percutaneous transcatheter coronary angioplasty to the right coronary artery followed by antihypertensive medication. Additionally, she had chronic kidney disease stage 3a, a history of pulmonary Koch, and fibrocavitatory changes as reported in a previous CT scan of the thorax. Her SpO₂ on room air was found to be 89%-90%, not improving with deeper inspiration. A recent ECHO showed an ejection fraction of 40%, left ventricular (LV) hypertrophy, basal-to-mid septal hypokinesia, and LV diastolic dysfunction grade 2, along with degenerative stenotic changes in the aortic valve, indicated by a peak velocity of 3.2 m/s, a mean pressure gradient of 29 mmHg, and a valve area of 1.3 cm². The mitral valve also showed degenerative ring calcification and moderate regurgitation due to coaptation failure. The patient was prescribed metoprolol XL 50 mg once daily, sacubitril-valsartan 50 mg twice daily, and Ecosprin 75 mg once daily. She has been oliguric while on diuretics and sodium bicarbonate tablets, and she was under regular follow-up with strict fluid and water management. She had completed antitubercular treatment (ATT) for six months. The surgery was performed after withholding sacubitril-valsartan for 48 hours.

Case 4

**Figure 4:**
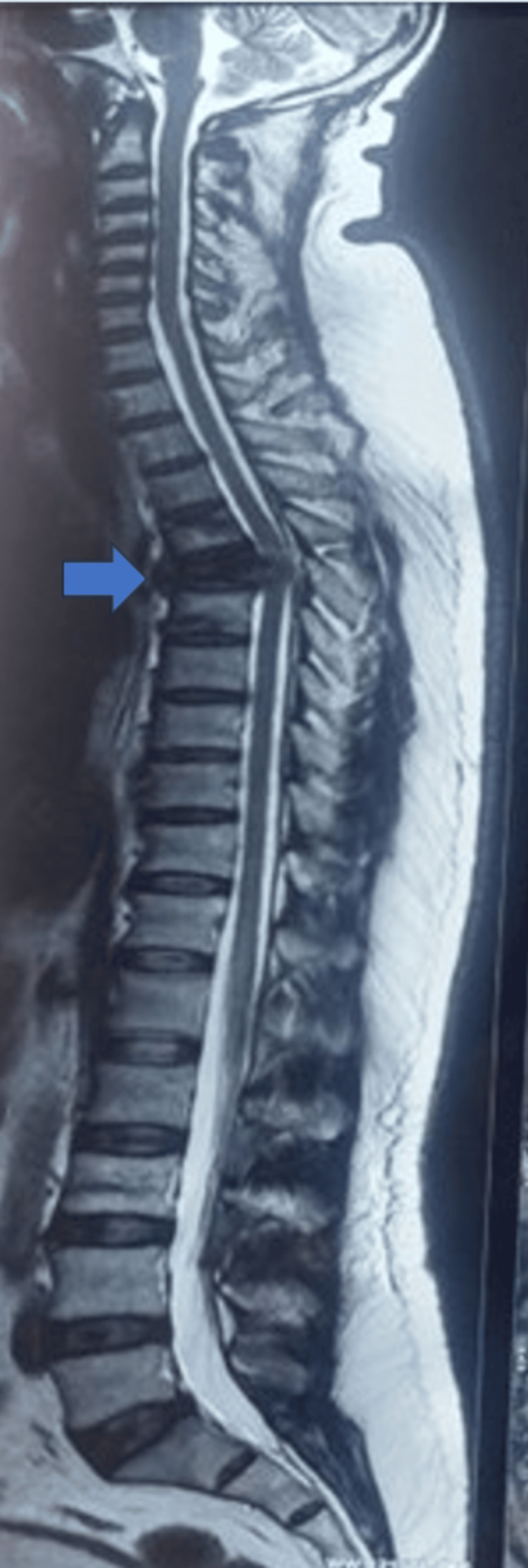
T6–7 fracture in an obese patient The blue arrow highlights the fracture site

A 32-year-old man with obesity presented with T6-7 fractures (Figure 4), along with a posterior rib fracture. He maintained saturation up to 90%-91% while receiving 4-6 L per minute of oxygen with a face mask. The patient had an abdominal girth of 100 cm and a body mass index of 32 kg/m². The ECHO showed moderate-to-severe LV hypertrophy and grade 1 LV diastolic dysfunction. The patient had significant dyspnea on exertion, classified as New York Heart Association (NYHA) class 2.

Case 5

**Figure 5:**
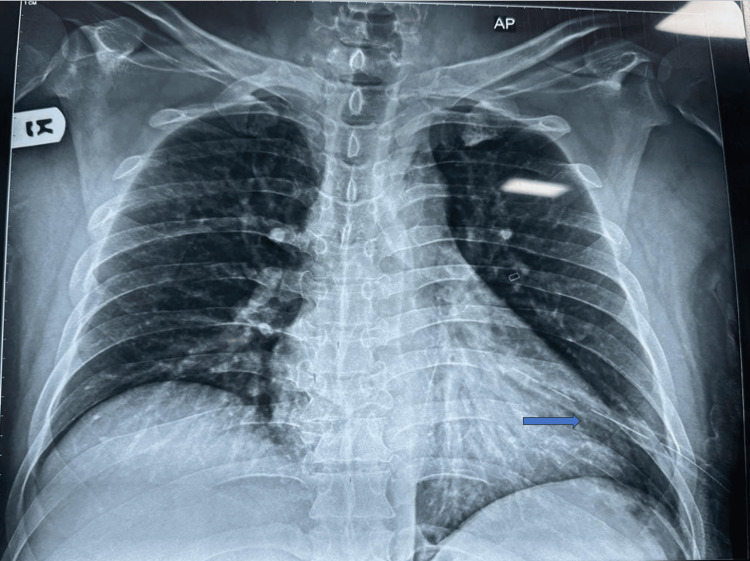
X-ray chest showing left intercostal drain (ICD) in place The blue arrow shows the intercostal drain

A 44-year-old male patient was scheduled for T3-5 fixation due to a fracture at the T4 spine. He had a history of hypertension, which was managed with amlodipine 10 mg once daily. His oxygen saturation was maintained at 89%-90% on room air. A left ICD was placed due to left-sided hemopneumothorax (Figure 5).

Anesthesia technique

The following describes the TSSA approach used across cases. All the patients were made to lie in a lateral position using a standard log-rolling maneuver with manual stabilization to ensure that there was no twisting, curling, or rotating of the spine [11]. Standard ASA monitors, including noninvasive blood pressure, five-lead ECG, pulse oximetry, and a temperature probe, were applied.

TSSA was performed by an anesthesiologist with over 10 years of experience in neuraxial anesthesia, using a 26-gauge Quincke needle at the specified thoracic interspace based on the surgical site (two levels below the lowest proposed site of fixation). A 1.8-2 mL dose of 0.5% isobaric levobupivacaine with 5 μg dexmedetomidine was injected over 30 seconds, ensuring no paresthesia. Ultrasound guidance was used in Case 4 due to obesity. The patients were positioned prone after five minutes, with their hands placed upward, maintaining the monitors and intravenous lines throughout the positioning. The level of sensory blockade was checked using standard pinprick stimuli. Once an adequate upper level of sensory block was achieved, the surgery commenced.

All the patients had intact hand movements assessed by the epidural scoring scale for arm movement (ESSAM). In all cases, no sedation was used. After securing the prone position, a bilateral erector spinae block with levobupivacaine 0.125% was administered. The postoperative pain relief was undertaken by the multimodal analgesia, with injection of paracetamol (1 g), tramadol (50 mg), and diclofenac (75 mg). Unlike other fractures, it may not be feasible or effective to apply a cast or splint for spine fractures.

All patients were counseled about the need to lie prone before receiving anesthesia. Each patient received an injection of ceftriaxone 1.5 mg intravenously and ondansetron 8 mg intravenously before the induction of anesthesia. An intravenous injection of dexamethasone (8 mg) was given at the time of laminectomy.

No major complications were noted in any of the cases. There was no need for conversion to GA, and the patients did not require additional analgesic or sedative medications intraoperatively. Hypotension was observed in Cases 1 and 4, which was managed with a single bolus of intravenous administration of 6 mg mephentermine with no further hemodynamic instability. Case 3 had bradycardia, dropping to 38 beats per minute, which was treated with an injection of 0.6 mg of atropine. None of the patients experienced any postoperative nausea and vomiting.

The duration of the surgery ranged from 90 to 120 minutes, whereas the duration of the block was 180-210 minutes. At the end of the surgical procedure, the patients were shifted to the postanesthesia care unit, where they were monitored for at least four hours before being transferred to the surgical intensive care unit. Oral sips were allowed after four hours, and all patients were given a soft diet for the night.

The postoperative course was uneventful in all cases; no patient required mechanical ventilation or deterioration of neurological consequences related to SA. As of the last follow-up date, all the patients were alive and maintained the functional and neurological status noted at discharge, which occurred at 30, 29, 27, 28, and 13 months after surgery.

## Discussion

This case series provides a preliminary report of TSSA in thoracic spine fractures, expanding its application beyond lumbar cases. Upper and mid-thoracic fractures are common in high-energy RTAs or in patients with a history of falls. It was found that the incidence of pulmonary complications was higher in patients with high thoracic injuries than in those having injuries in the lower thoracic region [6]. One case series reported that associated injuries include rib fractures leading to unilateral or bilateral hemothorax in 51% of cases or pneumothorax in 49%. Additionally, these patients may also have injuries to limbs in 28% of cases. These patients presented with neurological deficits, especially paraplegia in 60%, incomplete paraparesis in 23%, and intact neurology in 7% of cases [12].

The main concern is the deterioration of pulmonary function 24-72 hours after the injury, which occurs through three primary mechanisms. First, pain due to a fracture at the mid-thoracic spine leads to discomfort during deep breaths or coughing, leading to sputum retention and increased risk of pneumonia, especially in patients with rib fractures. This leads to hypoventilation and atelectasis, leading to the formation of pulmonary consolidation, a significant risk factor for GA. Again, associated pulmonary contusions cause impaired alveolar gas exchange, followed by progressive hypoxemia and hypercarbia. Restrictive lung diseases such as pneumothorax or hemothorax further exacerbate these conditions.

In our case series, one patient had bilateral hemothorax, two patients had unilateral hemothorax, one had a flail chest, and one had chronic renal failure with cardiac dysfunction. Owing to a high risk of developing cardiopulmonary complications during or after GA, as well as the elevated risk for mechanical ventilation after surgery, we planned to use TSSA. Although this approach is unconventional and less popular, it was deemed fit for this subset of patients [13]. The advantages of minimal hemodynamic fluctuations, early recovery, and less postoperative nausea and vomiting, especially in patients with respiratory comorbidities, are in favor of utilizing TSSA [14].

A recent study found a significant decrease in dynamic compliance and a significant increase in airway pressure during GA for spine surgeries performed in the prone position using a spine frame [15]. The study compared these findings with patients undergoing urological surgeries without a frame. However, either a spine frame or high bolsters are necessary for spine surgeries to ensure proper positioning.

GA for patients with respiratory involvement, such as rib fractures, hemothorax, or pneumothorax, prompts clinicians to reconsider their approach, especially when surgery involves pedicle screw fixation at the thoracic level. TSSA was reported as an alternative to GA in patients with ventricular dysfunction for thoracic spine surgery [16].

TSSA was first used by Jonnesco in 1909 and continues to widen its scope [17]. In 2005 [18], this technique was used for laparoscopic cholecystectomy in a patient with severe lung disease. A few published studies conducted in an MRI suite showed ample space between the spinal cord and posterior dura at the mid-thoracic level, indicating a potential area for subarachnoid drug instillation [19,20]. TSSA has been safely used in patients undergoing laparoscopic cholecystectomy, and it has proven to be a safer alternative to conventional lumbar SA [21].

An article published on surgeries at the thoracolumbar junction using double spinal needles, one at T5 and another at lumbar levels [22,23], found this technique to be adequate for pedicle screw fixation in terms of muscle relaxation, bolster tolerance, and patient comfort. This technique effectively blocked the entire spine from the upper thoracic level to the sacrum. TSSA was successfully used for scapular fracture [24].

Literature supports regional anesthesia as an effective alternative to GA, offering benefits such as lesser blood loss, improved outcomes, and reduced hospital stays. Additionally, it reduces the risk of prone position-related injuries such as ulnar nerve injury and face, eye, and brachial plexus injuries during lumbar spine fixation. Although regional anesthesia has not been used for upper thoracic fractures, no enhanced recovery after surgery (ERAS) protocol has yet been established for spine surgeries [25].

All patients in our case series were classified as very high risk for GA due to a high probability of requiring continuous postoperative elective mechanical ventilation in these cases. TSSA proved to be a lifesaver. All patients who consented to TSSA tolerated surgeries well, without any major side effects. They were able to resume feeding early and did not require postoperative mechanical ventilation for their chest conditions.

Limitations of the study include a limited surgical time window (180-210 minutes) provided by TSSA and the need for conversion to GA in long-duration surgeries. The absence of a control group and small sample size (n = 5) limit generalizability. Future studies should explore individualized dosing strategies based on spinal segment requirements and patient-specific factors.

## Conclusions

TSSA is a previously unexplored technique that involves blocking only specific segments using mid- or low-thoracic punctures. This technique can provide access to truncal surgeries under targeted patient-tailored neuraxial anesthesia safely. Perioperative hemodynamic stability, limited postoperative complications, and good patient satisfaction are notable advantages of the TSSA technique. However, larger multicentric trials are required to prove significant benefits and improve the acceptance of TSSA as a patient-first approach for truncal surgeries, especially spine surgery in moderate- to high-risk individuals.

## References

[1] Arshad MM, Khan MM, Al Sulaiti G, Al Rumaihi G (2024). Upper thoracic spine (D2-D3) fracture with unilateral lock facets without associated neurological deficits: case report & literature review. Int Med Case Rep J.

[2] el-Khoury GY, Whitten CG (1993). Trauma to the upper thoracic spine: anatomy, biomechanics, and unique imaging features. AJR Am J Roentgenol.

[3] Xing D, Chen Y, Ma JX (2013). A methodological systematic review of early versus late stabilization of thoracolumbar spine fractures. Eur Spine J.

[4] Marré B, Ballesteros V, Martínez C (2011). Thoracic spine fractures: injury profile and outcomes of a surgically treated cohort. Eur Spine J.

[5] Cicvarić A, Glavaš Tahtler J, Turk T (2024). Ventilation management in a patient with ventilation-perfusion mismatch in the early phase of lung injury and during the recovery. J Clin Med.

[6] Cotton BA, Pryor JP, Chinwalla I, Wiebe DJ, Reilly PM, Schwab CW (2005). Respiratory complications and mortality risk associated with thoracic spine injury. J Trauma.

[7] Meng T, Zhong Z, Meng L (2017). Impact of spinal anaesthesia vs. general anaesthesia on peri-operative outcome in lumbar spine surgery: a systematic review and meta-analysis of randomised, controlled trials. Anaesthesia.

[8] Perez-Roman RJ, Govindarajan V, Bryant JP, Wang MY (2021). Spinal anesthesia in awake surgical procedures of the lumbar spine: a systematic review and meta-analysis of 3709 patients. Neurosurg Focus.

[9] Khan IA, Siddiqui NH, Ramachandra SS, Nair A (2025). Indications and technique for thoracic segmental spinal anesthesia in clinical practice: a narrative review. Cureus.

[10] Paliwal NW, Khan IA (2025). Enhancing the safety of thoracic segmental spinal anaesthesia: do's and don'ts. Indian J Anaesth.

[11] Suter RE, Tighe TV, Sartori J, Reed K (1992). Thoraco-lumbar spinal instability during variations of the log-roll maneuver. Prehosp Disaster Med.

[12] Gattozzi DA, Friis LA, Arnold PM (2018). Surgery for traumatic fractures of the upper thoracic spine (T1-T6). Surg Neurol Int.

[13] Khan IA, Paliwal NW (2025). Challenges in implementing thoracic segmental spinal anesthesia in routine anesthesia practice. SBV J Basic Clin Appl Health Sci.

[14] Paliwal NW, Khan IA (2025). Thoracic segmental spinal anaesthesia: expanding applications while keeping it safe. Br J Anaesth.

[15] Kandasamy P, Pujari VS, Channaiah SR (2023). Effect of spine frame on the changes in respiratory dynamics in prone patients under general anaesthesia- a prospective, observational study. Indian J Anaesth.

[16] Vattipalli S, Lingareddy V, Chavali S, Singh S (2025). Thoracic segmental spinal block as an alternative to general anesthesia in patients with ventricular dysfunction for thoracic spine surgery. J Anaesthesiol Clin Pharmacol.

[17] Jonnesco T (1909). Remarks on general spinal analgesia. Br Med J.

[18] van Zundert AA, Stultiens G, Jakimowicz JJ, van den Borne BE, van der Ham WG, Wildsmith JA (2006). Segmental spinal anaesthesia for cholecystectomy in a patient with severe lung disease. Br J Anaesth.

[19] Lee RA, van Zundert AA, Breedveld P, Wondergem JH, Peek D, Wieringa PA (2007). The anatomy of the thoracic spinal canal investigated with magnetic resonance imaging (MRI). Acta Anaesthesiol Belg.

[20] Chandra R, Misra G, Pokharia P, Singh PK (2024). Study of thoracic spinal canal in Indian population with the 3.0 Tesla magnetic resonance imaging: exploring the safety profile of thoracic spinal anesthesia. J Anesth Clin Res.

[21] Imbelloni LE (2014). Spinal anesthesia for laparoscopic cholecystectomy: thoracic vs. lumbar technique. Saudi J Anaesth.

[22] Chandra R, Pullano C, Misra G, Agrawal P, Singh A (2023). Double needle technique-a novel approach of anaesthesia for thoracolumbar spine fractures. Austin J Anesth Analg.

[23] Chandra R, Pullano C, Khan IA (2025). Spinal anesthesia using the double-needle technique for thoracolumbar spine fracture surgery: a case series. Cureus.

[24] Dharamkhele SA, Nasre AH, Rathi MM, Gollapalli VK (2025). Thoracic segmental spinal anaesthesia for scapular fracture: a case report. Indian J Anaesth.

[25] Dietz N, Sharma M, Adams S (2019). Enhanced recovery after surgery (ERAS) for spine surgery: a systematic review. World Neurosurg.

